# Advice to the US FDA to Allow US Pharmacopeia to Create Biological Product Specifications (BPS) to Remove Side-by-Side Analytical Comparisons of Biosimilars with Reference Products

**DOI:** 10.3390/pharmaceutics16081013

**Published:** 2024-07-30

**Authors:** Sarfaraz K. Niazi

**Affiliations:** College of Pharmacy, University of Illinois, Chicago, IL 60015, USA; sniazi3@uic.edu

**Keywords:** biosimilars, FDA, US Pharmacopoeia (USP 2024), biological product specification (BPS), novel monograph, side-by-side reference product testing, validated analytical methods

## Abstract

Pharmacopeia monographs are not intended to establish biosimilarity. However, the US Food and Drug Administration (FDA) has stopped the US Pharmacopeia (USP) from creating monographs for biological drugs due to the need for side-by-side comparisons with the reference products. The USP can create Biological Product Specifications (BPS), not to be labeled as monographs, based on the analytical testing of reference products and validated test methods that will remove the need for side-by-side analytical testing of biosimilars with reference products. Scientific arguments confirm that this plan is logical and capable of creating global quality standards for biosimilars to allow their interchangeability with other biosimilars. While the regulatory agencies have waived many high-cost biosimilar tests, analytical assessment is the most sensitive test; reducing its cost will further enhance the entry of biosimilars with no clinically meaningful difference.

## 1. Introduction

**Figure 1:**
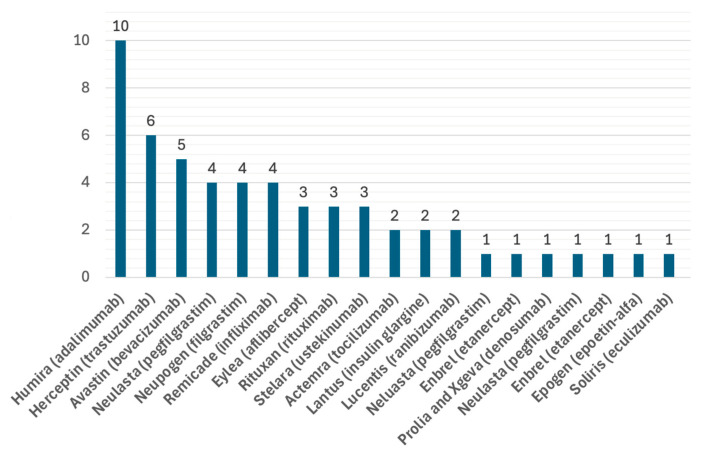
FDA-licensed biosimilars by the end of July 2024.

Since its passage of H.R. 3590 in 2009, the US Food and Drug Administration (FDA) has licensed 56 biosimilar products comprising 17 molecules [1] (Figure 1) out of more than 200 biological therapeutic proteins licensed by the FDA [2]; of these, more than half have expired patents that could be targeted as biosimilars [3].

Fifteen years after the passage of a bill to introduce biosimilars in the US, biosimilars remain inaccessible due to a lack of market competition caused by the high cost of their development, despite many recent changes in the regulatory requirements, such as waiving animal toxicology testing, granting interchangeable status without switching and alternating studies, and waiving efficacy testing when pharmacodynamic comparisons are available. While it is anticipated that the FDA will follow the examples set forth by the Medicines and Healthcare Products Regulatory Agency (MHRA), the MHRA has approved the biosimilars Ongavia and Vegzelma with clinical efficacy trials, relying instead on comprehensive analytical and pharmacokinetic data [4]. The European Medicines Agency (EMA) [5] is getting ready to adopt similar guidance as that provided by the MHRA [6]. In October 2023, the FDA announced a plan titled “Enhancing Adoption of Innovative Clinical Trial Approaches” [7], culminating in a conference in March 2024 [8] aimed at discussing strategies to enhance the efficacy and utility of comparative clinical trials. The plan emphasizes trial infrastructure, organizational culture [8], patient centricity, regulatory and compliance considerations, and the adoption of digital health technologies [9]. The FDA has also developed a portal [10] to gather stakeholders’ comments and suggestions to reduce redundant testing, potentially lowering drug development costs, particularly for biologicals. This study focuses on the contributions stakeholders can make to the FDA portal.

While the above evolution of regulatory guidelines will have a significant impact on the availability of biosimilars due to the reduced cost of their development, the most considerable test to establish biosimilarity, the analytical assessment, remains archaic and in need of a fresh evaluation, since this test is the most critical in establishing biosimilarity.

The US licensing of a product as a biosimilar or an interchangeable biosimilar [11] is based on the Biologics Price Competition and Innovation Act of 2009 (BPCI Act), which defines and mandates the information required to license biological products as biosimilar or interchangeable. The FDA has been proactive in rationalizing the biosimilar approval guidelines, bringing several changes as scientific rationality has grown with time. However, in the US, legislative actions are necessary for some changes, such as the interchangeable classification, the patent dance, or the selection of reference products.

Initially, the FDA enforced all stipulations of the BPCIA, as well as the guidelines derived from the BPCIA. Still, over time, as the knowledge and understanding of the safety and efficacy of biosimilars has been enhanced, the FDA has taken several actions as allowed in the BPCIA:

- The FDA withdrew a pivotal guideline for the analytical comparison [12] of biosimilars with their reference products using statistical modeling once it was demonstrated that the modeling was deficient in a citizen petition [13]; instead of revising the guideline, the FDA issued a new guideline [14] that notably changed the terminology from “comparison” to “assessment” and removed the challenged tier 1 statistical modeling and made the analytical biosimilarity testing more rational.
- The animal toxicology testing of biosimilars was listed in the BPCIA as one of the requirements; this was removed through a legislative change in the FDA Modernization Act 2.0 [15] that combined a previous US Senate bill [16] based on findings in the paper published in the *Science* magazine [17].
- The FDA interpreted the clinical efficacy testing requirement of the BPCIA as pharmacodynamic attributes, where available, such as erythropoietin, filgrastim, etc. [18,19]. This position of the FDA was questioned since the “omics” concept introduced by the FDA was considered impractical and irrelevant [20].
- In 2023, the FDA encouraged developers to create testing methods based on generally accepted scientific knowledge [21]; one recent change [22] on this basis was made by the FDA, allowing interchangeable status to biosimilars without additional switching and alternating studies that were questioned [23], while a Senate bill is in place to remove the interchangeable status of biosimilars from the legislation [24].
- In June 2024, the FDA proposed lectin-based assays [25,26] to compare the glycan profile of biological drugs, a more straightforward solution adaptable for high-throughput analysis [27]. The agency stressed that these comparisons need not be accurate if they are comparably resolved [28].
- The 21st Century Cures Act [29] includes a specific mandate for reference product companies to make their samples available to biosimilar developers. This mandate is part of the “Fair Access for Safe and Timely (FAST) Generics Act” provision within the Cures Act, requiring branded (reference product) companies to provide enough of their product to biosimilar and generic drug developers. This ensures that developers can conduct necessary testing to demonstrate bioequivalence and meet regulatory requirements. This provision aims to prevent anti-competitive practices, where reference product companies might refuse to supply their products, thereby hindering the development of more affordable biosimilars and generics. However, the implementation of this Act has failed as the branded companies have adopted various means of keeping their samples restricted from biosimilar developers by their availability and cost, as the developers need to secure multiple branded lots manufactured at different times. This exercise has become a critical hurdle in testing biosimilars.

## 2. Pharmacopeial Monographs

The FDA has been proactive in creative innovations [30], calling meetings to discuss biosimilar issues and secure stakeholders’ views. Regulatory agencies will go a long way in establishing a credible approach to making biological drugs affordable [31]. The FDA-EMA joint advisory program is an excellent example of such collaboration [32]. The pharmacopeias, the United States Pharmacopeia (USP), British Pharmacopoeia (BP), European Pharmacopoeia (EP), Japanese Pharmacopeia (JP), and International Pharmacopeia (IP), have played a pivotal role in the adoption of chemical generic products by creating thousands of monographs that specify the quality of products, creating a global quality standard.

The history of monographs for biological drugs in pharmacopeias dates to the early 20th century, when the need for standardized guidelines for biologic preparations became apparent. The United States Pharmacopeia (USP) introduced some of the earliest monographs for biological products, beginning with serum and vaccine standards in the 1900s. This effort was crucial in establishing consistent quality, safety, and efficacy criteria for biological drugs, which are inherently more complex than small molecule drugs. In 1942, the USP established the Biologics Division, which significantly expanded the inclusion of biological monographs. The European Pharmacopoeia (Ph. Eur.), which began in 1964, also incorporated monographs for biological products early in its history, setting standards for vaccines, blood products, and, later, biotechnologically derived medicines. These efforts were essential for harmonizing biological product standards across different regions, facilitating global trade and regulatory approval processes. These monographs’ development and continuous updating reflect advancements in scientific understanding and manufacturing technologies for biological drugs, ensuring they meet rigorous safety and efficacy standards [33].

Including recombinant product protocols in pharmacopeias marked a significant evolution in the standardization of biological drugs. In the 1980s, with the advent of recombinant DNA technology, pharmacopeias began to develop specific monographs and general chapters addressing these innovative products. The USP included detailed protocols for recombinant product production, characterization, and quality control. These protocols encompass a range of critical aspects, such as cell line development, fermentation processes, purification methods, and rigorous analytical testing to ensure product consistency, purity, and potency.

The USP General Chapter <1045> “Biotechnology-Derived Articles” provides comprehensive guidelines for developing and validating recombinant products, including monoclonal antibodies and therapeutic proteins. This chapter outlines essential protocols for the genetic stability of the production cell line, the control of the production process, and in-process testing. It also addresses the importance of post-translational modifications, such as glycosylation, which can significantly affect the efficacy and safety of the final product.

Similarly, the European Pharmacopoeia (Ph. Eur.) introduced monographs and guidelines for recombinant DNA products, which included specific tests for host–cell protein and DNA impurities, potency assays, and structural characterization. The Ph. Eur. also emphasizes the importance of validating the production process and establishing reference standards for recombinant products.

These protocols ensure that recombinant products meet stringent regulatory requirements and provide therapeutic benefits with high safety margins. The collaborative efforts of pharmacopeias worldwide have facilitated the global harmonization of standards, thus supporting the development and approval of safe and effective recombinant biological drugs.

Pharmacopeia monographs have long served the need to standardize testing methods and product specifications. However, these monographs are unsuitable for establishing a biosimilar product’s analytical similarity to its reference product. For example, the European Pharmacopoeia (Ph. Eur. 2023) sets public standards by providing harmonized quality requirements. However, a comparison of the biosimilar to a publicly available standard (e.g., a pharmacopeial monograph) is insufficient for comparability. Similarly, the reference standards described in Ph. Eur. monographs are not intended to be used as reference medicinal products (comparators) to demonstrate biosimilarity [34]. Table 1 lists the current monographs presented in the EP.

In addition, the European Pharmacopoeia has issued several general chapters, such as “Glycan analysis of glycoproteins” (2.2.59), “Abbreviations: Host–cell protein assays” (2.6.34), “Quantification and characterization of residual host–cell DNA” (2.6.35), and “Cell-based assays for potency determination of TNF-alpha antagonists” (2.7.26) that can be instrumental in creating the product monographs. The British Pharmacopoeia 2024 also contains similar monographs for recombinant drugs.

## 3. The FDA Stops Monographs

While the USP 2024 includes several monographs for biological drugs, its trailing off of adding more monographs and recombinant drugs began arriving with FDA approval when the FDA wrote to the USP in May 2018 [36], instructing that it should refrain from producing monographs for biological medications.

“Because USP’s proposed revisions would aggravate existing concerns that a monograph could impede or delay the licensure of biosimilars and other biological products, FDA strongly encourages USP to withdraw its proposal. FDA welcomes future interaction with USP on these issues to ensure that biological product monographs do not create an unnecessary barrier to the availability of biosimilars and other biological products to patients. For example, we see opportunities for optional methodological standards that could encourage innovation and product development.”

The FDA was concerned that biologic manufacturers would manipulate the monograph procedure to prevent competition from biosimilars “by incorporating patented characteristics of their product that are not relevant to safety, purity or potency, further impacting competition.” This demand by the FDA has left not just the USP but other pharmacopeias to add monographs that could be used in developing biosimilars.

## 4. Biological Product Specification (BPS)

Next to the clinical efficacy testing, a comparative analytical assessment of biosimilars with the reference products is the item with the highest cost and time requirements. While the FDA has rationalized specific testing requirements [12], there are no recommended protocols for specific biosimilar classes that can be adopted to reduce or remove the side-by-side testing of a biosimilar candidate with its reference product. The current test requires the collection of samples of the reference product throughout its expiry to ensure that the variability of the reference product is evaluated. In addition to the enormous cost, collecting reference product batches with different expiration dates is challenging, despite the Cures Act [29] and the Red Tape Elimination Act [37], which were supposed to remove this constraint.

One argument by the USP, when developing monographs, was the possibility that the FDA could share the release specification of the reference product; however, this information remains confidential, and these data are always dependent on testing methods. The biosimilar developers will have different testing methods, leading to inevitable side-by-side testing with the reference product.

However, these hurdles can be overcome if the FDA retracts its instructions and allows the USP to establish release specifications of the reference product, following the same approach as biosimilar developers, in side-by-side testing with commercially available multiple lots of the reference product. The USP can also provide validated test methods for all release specification releases that will eliminate the need for side-by-side testing. These specifications can also include potency tests that conform to the secondary and tertiary structure without using other biophysical test methodologies. The primary structure is determined by using peptide mapping, a method that can be validated, removing any further need for side-by-side testing. The USP can keep the BPS current as the reference product changes its formulation.

The FDA is concerned that if the USP borrows testing method details of specific methods that are the intellectual property of the referenced product, this will hinder the adoption of these test methods. These FDA concerns are ill-founded, since the USP has the technical expertise to develop non-infringing methods. The USP can also provide reference materials for qualification in analytical methods.

The above proposal is practically and scientifically sound and should bring no concern from the FDA or the USP. It is important to realize that these BPS are not creating monographs that might be confused with other monographs where a product is characterized independently; a better choice is to call it a BPS instead of a monograph.

## 5. BPS Attributes (BPSA)

**Figure 2:**
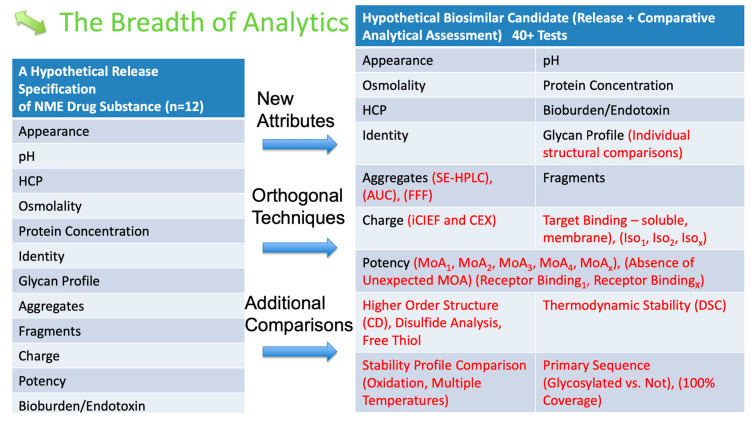
FDA-proposed testing of biosimilars to establish analytical similarity [38]. Items in red show new suggestions by the FDA. Host–Cell Proteins (HCPs) are proteins produced by the host organism (often bacteria, yeast, or mammalian cells) used to produce recombinant proteins or biologics. HCP analysis is crucial to minimize these proteins in the final product to avoid immune responses in patients. Size-Exclusion High-Performance Liquid Chromatography (SE-HPLC) is a chromatography method that separates molecules based on size. It is used to analyze the molecular weight distribution of proteins and detect aggregates in biopharmaceuticals. Analytical Ultracentrifugation (AUC) is a technique that uses high-speed centrifugation to measure the sedimentation properties of particles in solution. It characterizes macromolecules according to proteins’ size, shape, and interactions. Field-flow fractionation (FFF) is a separation technique that separates and characterizes macromolecules, nanoparticles, and colloids based on their size and molecular weight. It is used in the analysis of complex biological samples. Imaged Capillary Isoelectric Focusing (iCIEF) is a technique used to separate proteins based on their isoelectric point (pI) within a capillary tube. This method is valuable for assessing the charge heterogeneity of protein therapeutics. Cation Exchange Chromatography (CEX) is an ion exchange chromatography where positively charged ions (cations) are separated based on their affinity to the negatively charged stationary phase. It is commonly used to purify proteins and analyze their charge variants. Mechanism of Action (MoA) refers to the specific biochemical interaction through which a drug substance produces its pharmacological effect. Understanding the MoA is crucial in the development and characterization of biological drugs. Differential Scanning Calorimetry (DSC) is a thermal–analytical technique for studying proteins’ thermal stability and folding/unfolding properties. It measures the heat flow associated with thermal transitions in a sample.

In developing the BPS, the USP must be concerned with what attributes should be included. What attributes will be acceptable to the FDA? What attributes will be dependent on the nature of the product? At this stage, the FDA is encouraged to take a pivotal role. However, given the legality of any comments made by the FDA that might bring challenges to the FDA decision-making, the best way to reach this conclusion can come from the Type 2 meetings, where the developers can secure the FDA’s concurrence for the BPS. A good idea of how the BPS will look can be drawn from the recent FDA publications and reports; one such detail is provided in Figure 2 to establish analytical similarity.

It is noteworthy that over time, the FDA and other regulatory agencies have suggested many irrelevant suggestions in establishing analytical assessments that were removed when the FDA removed its pivotal “analytical comparison” guideline and replaced it with an “analytical assessment” guideline. However, there is an unmet need to adopt a rational scientific approach in deciding what constitutes a critical quality attribute [39]. Good examples of what should not be a critical quality attribute are included in the release specification. The inclusion of protein content, potency, impurities, glycans, etc., is redundant as the critical quality attribute in the comparative analysis, since these attributes are always part of the release specification [40].

This collaboration between the FDA and the USP is critical in making this exercise worthwhile. However, historically, and perhaps legally, the FDA has established that it is never bound to any pharmacopeial monograph of any product; however, the novel BPS proposed here can be endorsed by the FDA.

## 6. Advantages

A significant impact of the USP-derived BPS will harmonize the testing of biosimilars. Currently, there is a wide range of tests, and the number of tests reported in the filing of biosimilars can be misleading, particularly when the developers mistake extrapolation for repetitive testing, which is a clarification that USP can provide. The test method validation is the key to the success of BPS; now, it is well-established that the USP can validate all BPS methods; these methods also help to establish the similarity of attributes that are not part of the BPS, since all these are related to the BPS. Additionally, several methods, such as amino acid sequence secondary and tertiary structure analysis, can be used without bringing in the reference product.

Biosimilars licensed based on USP-based BPS will be expected to be more like other biosimilars, and thus, eventually, they could be accepted as interchangeable, legally as in the US and otherwise elsewhere. This consideration is vital as newer analytical methods continuously enter the field, yielding results that may not be compared with other methods [41], confounding the differences between biosimilars. This similarity may also lead to the global registration of biosimilars across many regulatory agencies.

## 7. Conclusions

The USP and other pharmacopeias have long been active in resolving quality issues by providing product monographs that include specifications and test methods; however, the FDA has halted this model for biological drugs. The FDA’s concerns can be removed by creating a novel biological produce specification model, not calling it a monograph that establishes the release specification of a biological product, eliminating the need for side-by-side testing of a biosimilar candidate with its reference product.

It is also anticipated that if the FDA allows the USP to create BPS, other pharmacopeia will follow this trend, or other regulatory agencies may allow the use of the USP-created BPS, leading to the global approval of biosimilars.

However, for this proposition to succeed, the USP will need substantial funding on an ongoing basis; the stakeholders, including the associations supporting biosimilars and the developers, should fund these projects; the NIH and the FDA also have significant funding available that can be given to the USP 2024 as grants, and finally, the USP may charge a licensing fee for the developers to use its data. Thus, the BPS, not labeled as a monograph to avoid confusion with other monographs, can lead to a remarkable quality improvement of biosimilars.

## Figures and Tables

**Table 1:** European Pharmacopoeia biotherapeutics monographs [35]. Parentheses show the pharmacopeia monograph designation number.

Issued Monographs	New Monographs in Preparation
Alteplase for injection (1170) ^§^ Calcitonin salmon (0471) Erythropoietin concentrated solution (1316) ^§^ Etanercept (2895) Filgrastim concentrated solution (2206) Filgrastim injection (2848) * Follitropin (2285) Follitropin concentrated solution (2286) Glucagon, human (1635) Human coagulation factor IX (rDNA) powder for solution for injection (2994) * Human coagulation factor IX rDNA concentrated solution (2522) Human coagulation factor VIIa rDNA concentrated solution (2534) Human coagulation factor VIII rDNA (1643) ^§^ Infliximab concentrated solution (2928) ^§^ Insulin aspart (2084) Insulin glargine (2571) Insulin lispro (2085) Insulin preparations, injectable (0854) * Insulin, human (0838) Interferon alfa-2 concentrated solution (1110) Interferon gamma-1b concentrated solution (1440) Molgramostim concentrated solution (1641) Somatropin concentrated solution (0950) Somatropin (0951) Somatropin for injection (0952) * Somatropin solution for injection (2370) * Teriparatide (2829)	Alteplase concentrated solution (3197) Adalimumab (3147) Darbepoetin alfa (3009) Golimumab concentrated solution (3103) Golimumab injection (3187) * Human coagulation factor VIII (rDNA) concentrated solution (3105) Human coagulation factor VIII (rDNA) powder for injection (3106) * Human coagulation factor VIII (rDNA), B-domain deleted, concentrated solution (3107) Human coagulation factor VIII (rDNA), B-domain deleted, powder for injection (3108) * Insulin glargine injection (3129) * Pegfilgrastim (2889) Teriparatide injection (3130) * Ustekinumab (3165) Ustekinumab injection (3188) *

* Finished product monographs; ^§^ under revision.

## Data Availability

No new data were created or analyzed in this study.
